# Acute renal failure after dietary change – a case report

**DOI:** 10.1186/s12882-026-04906-0

**Published:** 2026-03-24

**Authors:** Eric Jankowski, J. Reindl, L. Hauptmann, T. Wiech, G. Wolf, M. Busch

**Affiliations:** 1 Department of Internal Medicine III, University Hospital Jena, Friedrich Schiller University, Am Klinikum 1, 07747 Jena, Germany; 2 Center for Diagnostics, Institute of Pathology, Section of Molecular Pathology and Cytopathology, University Medical Center Hamburg Eppendorf, Hamburg, Germany

**Keywords:** Oxalate nephropathy, Acute kidney injury, Swiss chard, Case report

## Abstract

**Background:**

The disease entity oxalate nephropathy (ON) is an uncommon but devastating cause of chronic kidney disease associated with the deposition of calcium oxalate crystals in the kidney tubules. The prognosis of ON is poor and targeted therapy is lacking.

**Case presentation:**

Here, we report the case of a previously kidney-healthy 59-year-old White female presenting with acute renal failure (ARF) with enlarged kidneys after the consumption of one meal of freshly harvested Swiss chard as part of a longer-term vegetarian dietary change. Kidney biopsy indicated acute tubular damage with intratubular obstruction by oxalate crystal deposition and no evidence of chronic kidney damage or other relevant pathologies. Under conservative therapy and a low-oxalate diet, kidney function improved and the kidneys sonomorphologically returned to a normal size 4 weeks later. After continuing a low-oxalate diet for one year, a return to baseline eGFR was observed, which highlights the importance of nutritional intervention in such cases.

**Conclusion:**

This case illustrates the importance of dietary patterns even in cases of an acute kidney failure. It raises relevant research questions, particularly regarding the threshold amount of oxalate crystal deposition in renal tissue that predicts clinically significant oxalate nephropathy.

## Background

Oxalate nephropathy (ON) usually describes chronic kidney disease resulting from calcium oxalate crystal deposition in kidney tubules. Acute tubular injury and associated interstitial nephritis or fibrosis are also found in such cases [1].

Oxalate is a naturally occurring dicarboxylic acid. As a product of incomplete carbohydrate degradation, oxalate occurs in plants. The homeostasis of oxalate in humans is a complex and finely regulated process involving supply, metabolism and excretion. Oxalate is partially absorbed in the small intestine. Only oxalate that is not bound to other ions, such as calcium, is efficiently absorbed and enters the bloodstream [2]. Oxalate excretion is performed by the kidneys and is determined by the glomerular filtration rate, tubular reabsorption and active secretion in the proximal tubule. In addition to exogenous oxalate, the liver is the primary source of endogenous oxalate synthesized from precursor glyoxylate, contributing to 60–80% of plasma oxalate. Hyperoxaluria can be separated into primary and secondary forms on the basis of the underlying disorders involved in the key steps of oxalate homeostasis [2].

Primary hyperoxaluria (PH) is a group of autosomal recessive disorders leading to hepatic overproduction of oxalate by enzyme defects in the glyoxylate metabolic pathway, resulting in calcium oxalate nephrolithiasis and oxalate deposition in other organs. PH most commonly is a pediatric disease but should also be considered in adults when an obvious cause of ON is missing [1, 2].

Enteric hyperoxaluria (EH) is a secondary form of hyperoxaluria caused by increased oxalate uptake from the gut and occurs in the context of fat malabsorption or steatorrhea [1]. Calcium normally binds oxalate in the bowel, forming insoluble calcium oxalate, which is excreted in the feces. In the case of fat malabsorption, calcium becomes unavailable due to increased binding of calcium to free fatty acids, resulting in increased soluble and thus absorbable oxalate. Furthermore, free fatty acids also directly increase the permeability of the colon to oxalate [3]. EH is usually not observed in healthy guts but is associated with bariatric surgery, exocrine pancreatic insufficiency, Morbus Crohn’s disease, *Clostridium difficile* infection, or the use of octreotide and orlistat [4]. In EH, the risk of renal precipitation of oxalate is worsened by hypovolemia and bicarbonate loss associated with diarrhea, leading to metabolic acidosis and hypocitraturia [1].

Another secondary cause of Oxalate nephropathy (ON) is the increased direct uptake of oxalate or oxalate precursors. A dramatic example of ingestion-induced ON is ethylene glycol intoxication, an ingredient of industrial antifreeze, solvents, paintings and other commercial products. The liver metabolizes ethylene glycol to oxalic acids, causing ON [5]. Fewer causes are shown by case reports of ON in the context of comorbidities and excessively increased uptake of high oxalate foods or oxalate precursor vitamin C, which is nonenzymatically metabolized to oxalate, especially for an increased chronic intake. Data on the contribution of dietary oxalate to urine oxalate excretion differ from 10 to 80% (6). In particular, ON associated with chronic intake of vitamin C supplements is reported frequently, likely due to a better bioavailable source of oxalate than food, where oxalate is complexed with calcium and magnesium (1).

## Case presentation

**Fig. 1:**
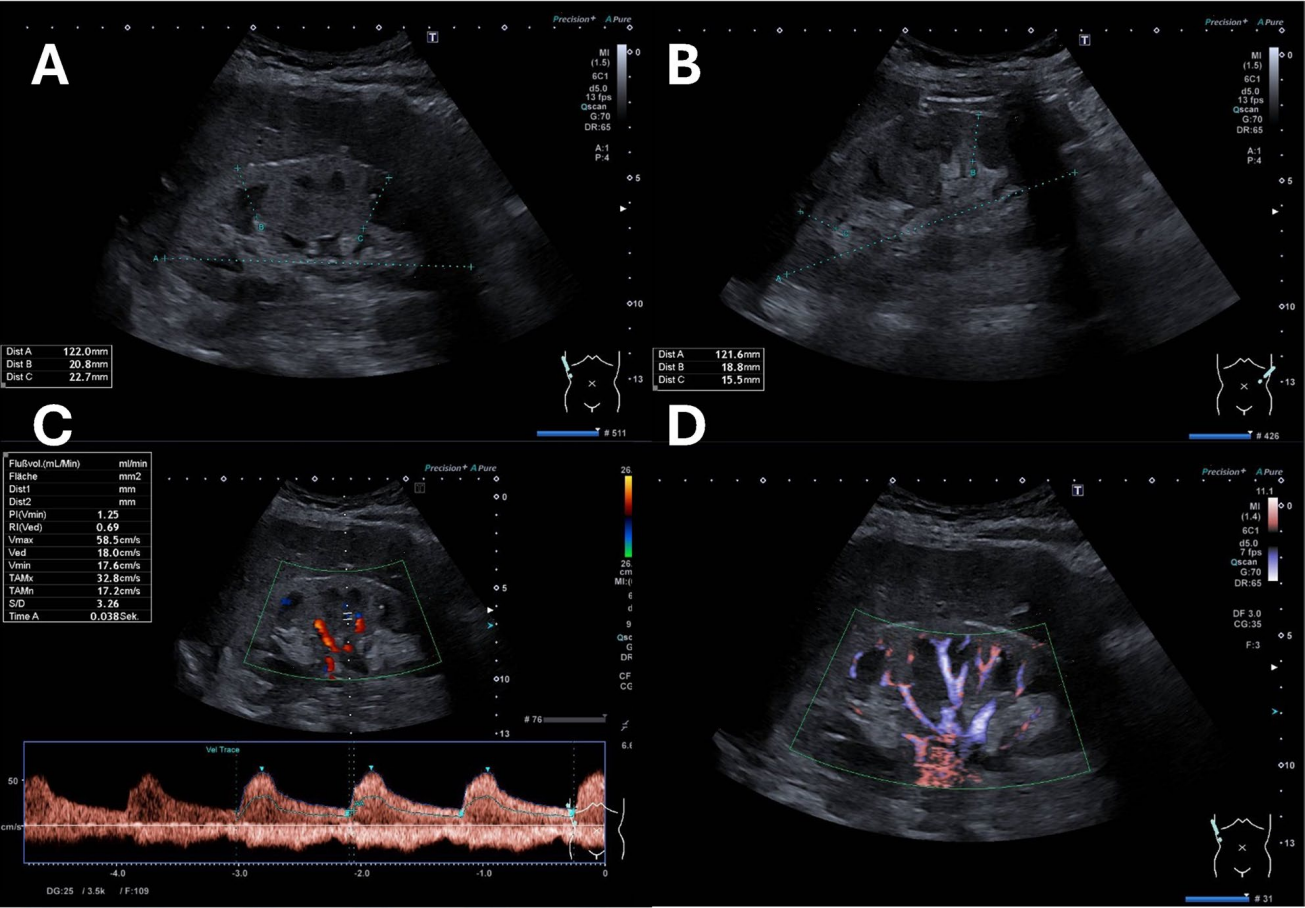
Duplex sonography at the time of admission showing enlarged and swollen kidneys with normal blood flow

A 59-year-old white female (body height: 163 cm; body weight: 80.2 kg) with prior stable kidney function while treated for arterial hypertension (March 18th at the general practitioner: serum creatinine 80 µmol/l; eGFR 70 ml/min/1.73 m²) was hospitalized on the 14th of September in a district hospital with dull, diffuse abdominal pain and intermittent stabbing pain originating from both flanks. She reported nausea with inappetence for 3 days. The clinical examination revealed diffuse pain on pressure throughout the upper abdomen and bilateral costovertebral angle (CVA) tenderness. An initial laboratory check revealed a significant increase in serum creatinine up to 412 µmol/l (eGFR 10 ml/min/1.73m^2^), with a blood urea nitrogen of 13,6 mmol/l. Postrenal renal failure was ruled out by ultrasound and infusion therapy with 1 L of a balanced crystalloid solution (comparable to Ionolyte or Plasma-Lyte 148^®^; containing physiologic concentrations of sodium, potassium, calcium, magnesium, and chloride, buffered with acetate) was administered, resulting in documented urinary excretion of 1300 ml. Given that renal retention parameters did not improve the next day, the patient was transferred to the university hospital. At admission, the physical examination revealed no obvious signs of volume depletion, with grade 1 hypertensive blood pressure readings (153/93 mmHg), a normal heart rate (65 bpm) and normothermia (36.6 °C). CVA tenderness or any pain was no longer reported. The patient’s home medication included therapy with an angiotensin-converting enzyme inhibitor (ACEi; ramipril 5 mg/d) and a calcium antagonist (amlodipine 5 mg/d) to treat arterial hypertension. The patient’s history revealed no evidence of nicotine consumption or alcohol abuse. The in-hospital laboratory test revealed persistently impaired kidney function, with a rise in serum creatinine of up to 407 µmol/l (eGFR 9.8 ml/min/1.73m^2^) and a blood urea nitrogen of 14.7 mmol/l. Dysuric complaints were denied. The C-reactive protein level was slightly elevated at 32,8 mg/l, but clinically, no signs of infection were detected. Proteinuria was found to be normal (spot-urine: 140 mg/gCrea; 24 h-urine sample: proteinuria < 40 mg/d; albuminuria 18 mg/d). The liver enzymes, as well as the serum-lipase and vasculitis serology, were normal (Table 1), whereas kidney function further declined on the following day (serum creatinine 421 µmol/l; eGFR 9.4 ml/min/1.73 m²). Sono-morphologically, the kidneys were clearly enlarged and swollen. A visible obstruction of the urinary tract could be excluded (Fig. 1). A general abdominal X-ray revealed slight coprostasis but no evidence of airfluid levels, signs of ileus or free air. The chest X-ray revealed no signs of acute cardiopulmonary congestion with a normal mediastinal configuration. There were also no pulmonary infiltrates or intrapulmonary lesions.

**Table 1:** Relevant laboratory values on the day of admission. Abnormal laboratory results are in bold type

Laboratory Results	At Admission	Standard Values
Sodium (mmol/l)	137	136–145
Potassium (mmol/l)	**4.37**	3.4–4.5
Calcium (mmol/l)	**1.36**	2.15–2.50
Creatinine (µmol/l)	**407**	44–80
eGFR (ml/min/1.73 m²)	**9.8**	> 90
Blood urea nitrogen (mmol/l)	**14.7**	3.5–7.2
C-reactive protein (mg/l)	**32.8**	< 5.0
ALAT (µmol/l*s)	0.11	< 0.58
ASAT (µmol/l*s)	**Haemolytic**	< 0.60
yGT (µmol/l*s)	0.13	0.10–0.70
LDH (µmol/l*s)	**Haemolytic**	< 4.2
Leucocytes (Gpt/l)	7	4.4–11.3
Haemoglobin (mmol/l)	**7.5**	7.6–9.5
Quick (%)	115	70–130
INR	0.9	
*Blood gas analysis*		
pH ven.	**7.33**	7.35–7.45
Oxygen partial pressure ven. (kPa)	**3.99**	4.8–5.87
Carbon dioxide partial pressure ven. (kPa)	5.65	4.93–6.67
Standard bicarbonate (mmol/l)	21.0	21–26
Akt. bicarbonate (mmol/l)	22.6	21–26
Base excess (mmol/l)	**-3.2**	-2.0–3.0
*Urine sediment*		
pH	**7**	5–6
Erythrocytes/µl	3	< 17
Leukocytes/µl	14	< 28
Bacteria/µl	**(+)**	Negative
*Urine protein profile from spot urine*		
Protein (mg/l)	< 40	< 150
Albumin (mg/l)	18	
Albuminuria (mg/gCrea)	**64**	< 20
a1-Microglobulin (mg/l)	< 5.0	

The detailed case history revealed a change in diet from the beginning of the year towards vegetarian eating habits, as shown in Table 2. Furthermore, the patient reported having eaten freshly harvested and cooked Swiss chard leaves in an amount of approximately 500 g first time ever the day before the onset of symptoms.

**Table 2:** Changes in eating habits since the beginning of the year

Meal	Eating habits
Breakfast	100–150 g of muesli (oats, spelt, barley flakes, buckwheat, hemp seeds, linseed, wheat bran, rosehips, prunes, amaranth) mixed with 100 ml orange juice
Lunch	Mainly vegetarian diet with various vegetables, often potatoes and fish every 2 weeks
Vespers	100 g of nuts, pureed garden fruits with milk or yogurt
Dinner	High-protein diet with various vegetables

Six months previously, the patient had in terms of age and under treatment with ramipril for arterial hypertension a slightly reduced kidney function with an eGFR of 70 ml/min/1.73 m². Owing to the rapid deterioration in kidney function with an unclear origin and no improvement by infusion therapy (Fig. 3), a kidney biopsy was performed on the 16th of September for further diagnostics.

**Fig. 2:**
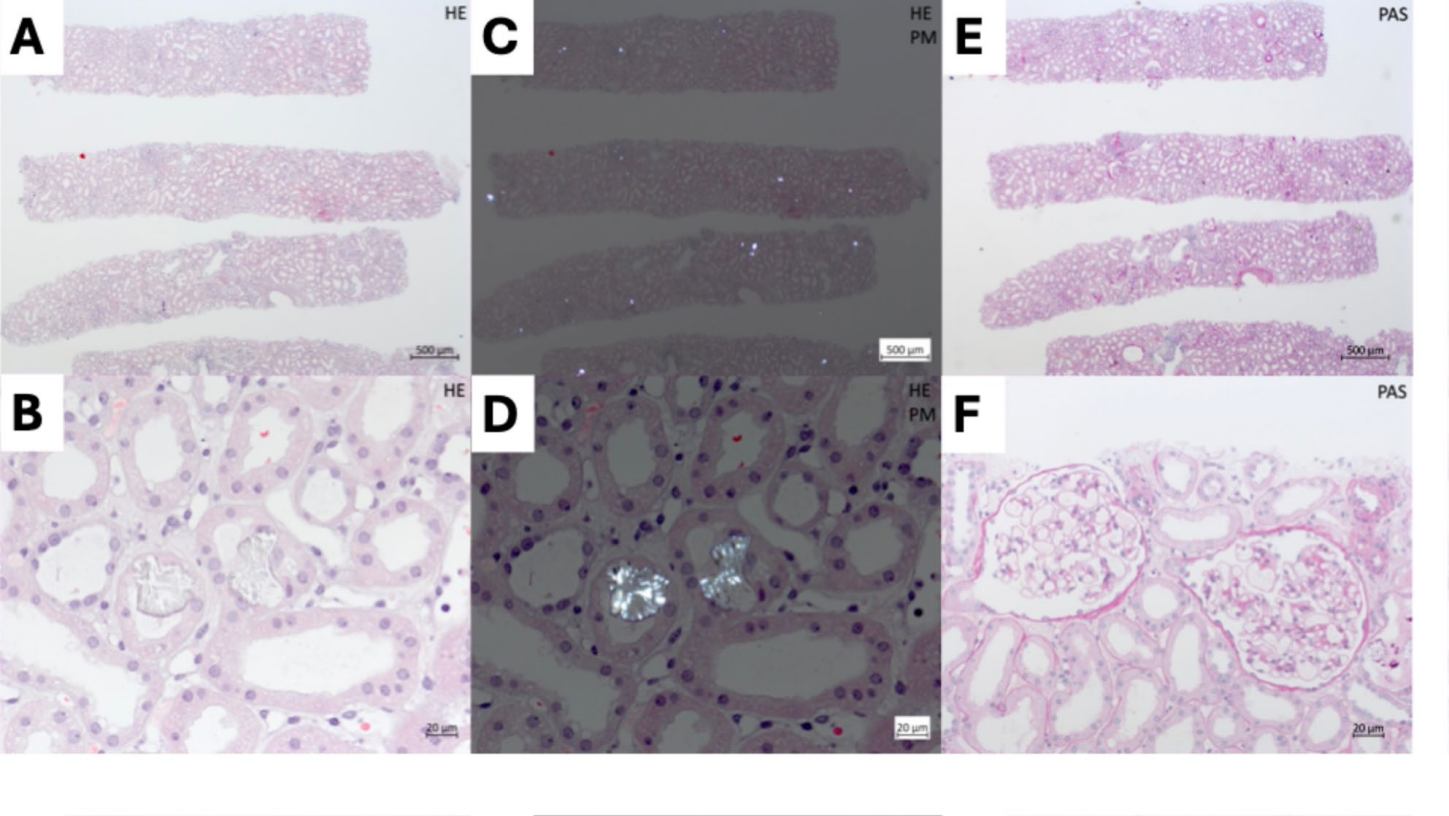
Light microscopy images showing moderate diffuse tubular damage and intralobular oxalate crystal deposition. **A + B** Hematoxylin and eosin staining; **C + D** Hematoxylin and eosin staining under polarized light; **E + F** Periodic acid–Schiff staining

The light microscopy findings revealed acute, diffuse, potentially reversible tubular damage to the cortex. Almost all the tubules were dilated, with a flattened epithelium and brush border defects. In several clear spaces in tubules, birefringent, Kossa-positive, partially fan-shaped microcalcifications compatible with oxalate crystal deposits were observed. No evidence for glomerulonephritis or interstitial nephritis was found (Fig. 2).

The amount of oxalate in a 24-h urine sample taken 7 days after the onset of symptoms was determined to be normal at 31 mg/l. Conservative therapy was carried out by means of moderate volume substitution with a balanced crystalloid solution (1000 ml/day for three days) and nutritional counselling with a strict low-oxalate diet from then on, which resulted in an improvement in kidney function (serum creatinine 243 µmol/l, eGFR 18.2 ml/min/1.73m^2^) only one week later. The patient then was discharged from the hospital.

**Fig. 3:**
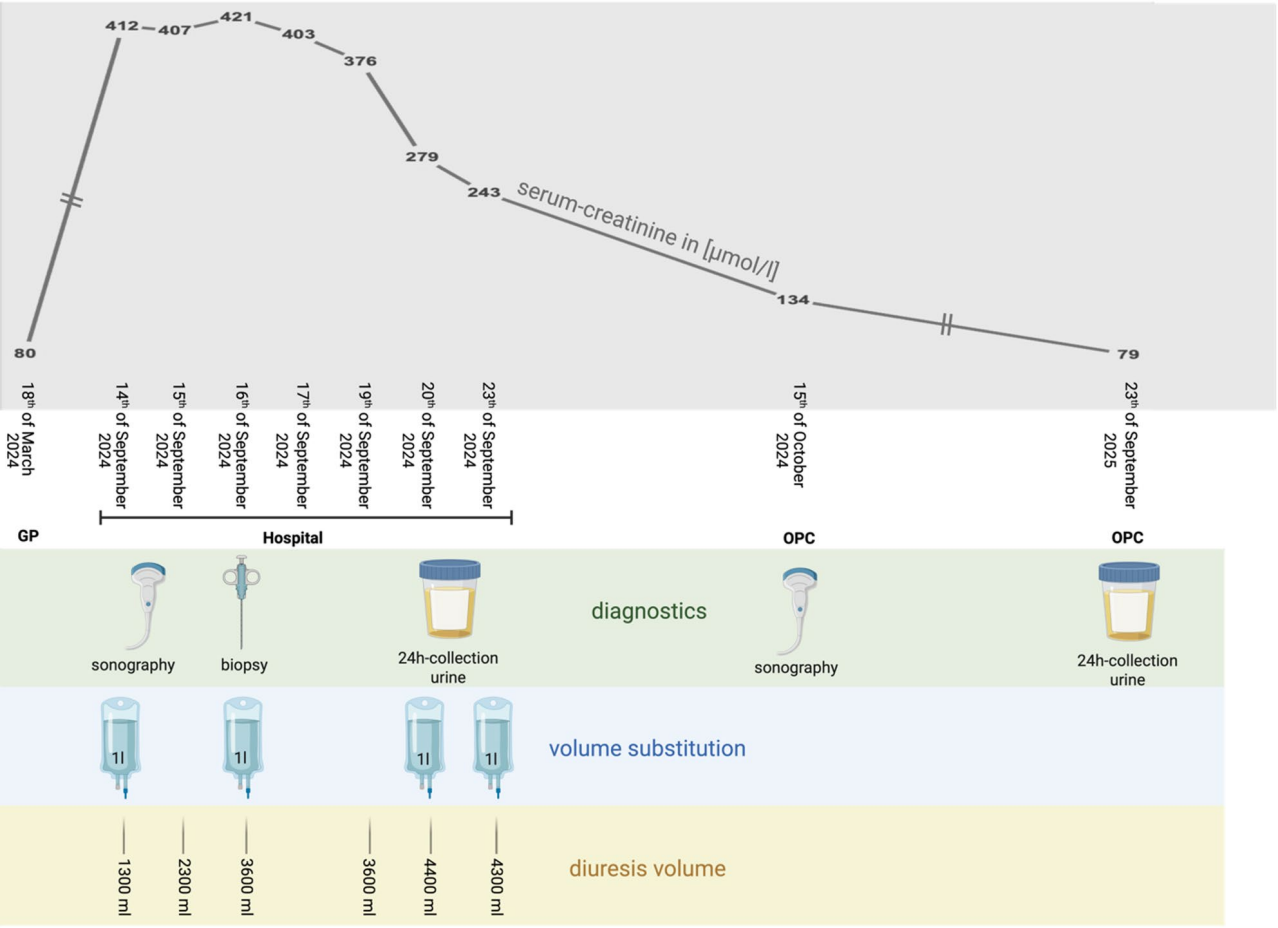
Illustration of the clinical course with reference to the diagnostics and volume substitution in relation to the course of serum creatinine. Infusion therapy was performed by using a full electrolyte mixture. The urine output per day was measured in ml. GP - General Practitioner. OPC - Outpatient Clinic. Figure created via Biorender

A follow-up at the outpatient clinic 4 weeks later revealed further improvement in renal function, with a serum creatinine of 134 µmol/l (eGFR 37 ml/min/1.73m^2^) together with a sonomorphological return to normal kidney size. The patient was informed about the necessity of a permanently oxalate-reduced diet. She was instructed to avoid foods high in oxalates (Table 3), even though she continued to eat a vegetarian diet. One year after discharge from the hospital (on the 23th of September 2025), the patient’s kidney function returned to the initial baseline value, with a normal serum creatinine level of 79 µmol/l and an eGFR of 70.4 ml/min/1.73m^2^. Interestingly, oxalic acid in the 24-hour urine sample at the same time was slightly elevated for the first time at 49 mg (normal < 45 mg/24 hours), suggesting that less oxalate is now crystallizing in the kidneys than at the time of acute oxalate nephropathy. In addition, there was a slightly elevated uric acid excretion of 6.05 mmol/24 h (reference range 1.2–5.9 mmol/24 h), consistent with a mild hyperuricosuria. Creatinine excretion in the collected urine was 17.7 mmol/24 h, confirming adequate 24-hour urine collection. The urine pH was 6.5 (reference range 5–6). Figure 3 shows the whole course of the patient, together with the diagnostic and therapeutic interventions.

**Table 3:** High-oxalate foods and amount of soluble oxalic acid depending on the method of preparation [7]

Food	soluble oxalic acid in mg/100 g fresh weight
Spinach	raw: 90–2000 boiled: 91–184 frozen: 615–1093
Rhubarb stalks	raw: 287 boiled: 81
Swiss chard leaves	raw: 252 boiled: 218
Sorrel	raw: 1079
Taro leaves	raw: 204–267
Amaranth	raw: 835

## Discussion

Although impressive, this case warrants discussion in relation to previously published reports on diet-related oxalate nephropathy. Although oxalate nephropathy is most commonly associated with chronic hyperoxaluria or other predisposing conditions, there are individual case reports describing acute excessive intake of oxalate-rich foods or oxalate precursors as potential triggers for acute ON such as star fruit, nuts, rhubarb, iced tea, or chaga mushrooms. To the best of our knowledge, there are currently no reports of acute ON in connection with the consumption of freshly harvested and cooked chard leaves.

Hyperoxaluria depends on the rate of intestinal absorption, bioavailability and the rate of degradation by bacterial flora [4]. Only approximately 15% of dietary oxalate is absorbed, and a chronic intake of 50–200 mg/day is considered to be safe [2].

The oxalate content varies between several vegetables and even between one type of vegetable on the basis of how it is prepared, the season in which it is harvested and between the leaf with the highest oxalate level and the root with the lowest oxalate level [6]. The occurrence of oxalate in foods, especially in green leafy vegetables, is relevant and some are considered high-oxalate foods [7] (Table 3).

The estimated preparation and intake of 500 g of fresh weight chard leaves reported in this case resulted in a calculated oxalate intake of approximately 1040–5415 mg in total and 1090 mg of soluble oxalic acid [7]. The exact amount of bioavailable oxalate ingested can be estimated only on the basis of the numerous influencing factors mentioned. Assuming a safe amount of 50–200 mg/day, the ingested amount still represents an excess, most likely contributing to the oxalate deposits found in the kidneys leading to acute kidney failure (Fig. 2).

However, the chronically elevated oxalate load resulting from the patient’s dietary changes since the beginning of the year (Table 2) must be considered as a plausible contributing factor. Especially for smoothies and juice diets, there are case reports of oxalate nephropathy [4, 8]. Accordingly, the daily consumption of pureed fruits and vegetables in addition to nuts for vespers in this case, as well as the switch to a morning muesli with amaranth since the beginning of the year, should be considered additional major factors alongside the patient’s generally oxalate-rich plant-based diet (Table 2). Persistently high intake of oxalate-rich foods can promote renal crystal deposition, thereby potentially lowering the threshold for clinically manifest kidney injury. In this context, the event of acute renal failure may reflect the “tip of an iceberg” of a cumulative process of oxalate deposition rather than an isolated dietary trigger. Given the short latency period of only one day between excessive chard intake and the onset of symptoms, and three days until a significant deterioration in eGFR was detected, a link between excessive oxalate intake from a meal containing chard and the observed acute renal failure is likely. The single intake of an excessive amount of oxalate through the consumption of a large quantity of Swiss chard leaves may therefore have acted as superimposed precipitating event that exceeded the compensatory capacity of the kidneys and ultimately led to apparent renal dysfunction. In this context, oxalate deposition is subject to a variety of mediating factors including supersaturation, precipitation and deposition. The crystallization process within the tubular fluid and urine is dependent on the solution composition. Oversaturation with oxalate is the driving force for crystal formation, and lower levels of urinary calcium and magnesium favour crystallization [2, 9, 10]. The conditions of volume depletion increase the precipitation of oxalate, which then leads to an obstructive tubulopathy associated with mild tubular damage, as observed in the case presented here.

Volume depletion due to nausea with a single episode of vomiting and reduced fluid intake possibly had a further impact on the severity of acute renal failure since the tubular precipitation of oxalate crystals is facilitated by an increase in urine concentration. However, the prolonged restitution of kidney function despite early volume substitution argues against volume depletion alone as the primary cause of the renal impairment, supporting its role as a contributory rather than determining factor.

The causes of chronic ON are mostly hyperoxaluria characterized by an increased 24-h urine oxalate excretion of > 40–45 mg/day [1]. Multiple studies have shown a higher excretion rate of oxalate in patients with diabetes mellitus than in those without [11]. In this case, the patient was not known to have diabetes mellitus, and the HbA1c was determined to be 5.1%. The initial amount of urine oxalate found in the patient described here was normal at 31 mg/24 h. Because there was a latency time of 7 days between the onset of symptoms and the 24-hour urine collection measurement, we believe that the acute chronic oxalate overload combined with mild volume depletion led to an exceedance of the solubility product for oxalic acid in the urine. In our opinion, the resulting precipitation of oxalic acid into oxalate crystals, which were evident in the histological examination, is one reason for the normal oxalic acid excretion at this acute stage. Increased oxalate excretion before exceeding the solubility product and causing acute ON can be assumed. However, no previous oxaluria measurements or other urinary parameters related to kidney stones were available for this patient since there was no need for it prior to the onset of the acute illness. Interestingly, slightly elevated oxalate levels were measured in the 24-hour urine sample one year after discharge (49 mg/24 h on the 23th of September 2025). The urine pH in the last follow-up measurement was with 6.5 slightly above the reference range (5–6). This pH value typically reduces the precipitation of uric acid, but still promotes the formation of calcium oxalate crystals. In summary with the slightly elevated uric acid excretion of 6.05 mmol/24 h (reference range 1.2–5.9 mmol/24 h), the last follow-up urine profile shows a slightly increased lithogenic environment with mild hyperoxaluria and mild hyperuricosuria, whereby uric acid may act as a possible nucleation factor for crystallization. In our view, this confirms our assumption that the amount of oxalic acid excreted in the urine over time returned to the urine-soluble product and can thus be detected as free oxalic acid. The patient was again advised to follow a low-oxalate diet and the findings were explained. Given the slightly increased lithogenic environment Follow-Up controls were scheduled.

Kidney biopsies from ON patients revealed crystalline nephropathy characterized by the precipitation of oxalate crystals in the kidney tubules under polarized light. Unlike phosphate crystals, calcium oxalate crystals are birefringent under polarized light [1]. The triggering of chronic interstitial kidney damage is likely due to a downstream inflammatory process induced by proinflammatory molecules released from crystal-damaged tubular cells. These proinflammatory molecules seem to recruit immune cells into the interstitium and activate local macrophages and dendritic cells [12]. To date, there is no nephropathologically proven crystal density in biopsies that can serve as an indicator of kidney failure. As in this case, the clinical picture is decisive.

There are recommendations for reducing the risk of developing crystal nephropathy by maintaining adequate volume status and avoiding the simultaneous use of diuretics, ACE inhibitors/ angiotensin II_1_ receptor blockers and NSAIDs [9, 10, 13]. In this context our patient`s preexisting ACEi therapy may have contributed to an increased susceptibility by lowering the threshold for renal injury, particularly in the setting of reduced volume intake and evolving oxalate burden. Accordingly, the medication was temporarily withheld at admission and was cautiously reintroduced at discharge after renal function recovered.

Treatment of ON has limited outcome data and should be tailored to the underlying causes. Whereas oral citrate is used to inhibit crystallization in primary and secondary hyperoxaluria, treatment of PH depends on the type of mutation and new drugs, such as lumisaran (an RNA interference agent that degrades the mRNA for hepatic glycolate oxidase), are being approved. For ON in the context of diet-induced hyperoxaluria, the identification and elimination of the oxalate source is crucial.

As this case report shows, educating the patient about her dietary habits and the oxalate content of the foods she had been eating more since the beginning of the year had a significant effect on her kidney function. Continuing with a low-oxalate diet led to a marked improvement in her kidney function just four weeks after her discharge from the hospital. After one year, we observed a return to the baseline eGFR.

Nonspecific treatment options furthermore include increasing calcium uptake for binding oxalate in the bowel, maintaining high fluid intake and lowering fat intake [1]. In cases of crystal nephropathy, sustaining a high urine flow rate is mandatory for minimizing further precipitation of obstructing oxalate crystals [13]. The use of Sevelamer hydrochloride to bind fatty acids or the administration of cholestyramine to reduce the effect of bile acid on colon permeability and direct oxalate binding has shown no significant results [1, 14]. Another therapeutic strategy involving manipulation of the microbiome with probiotics or nonpathogenic anaerobic bacteria, such as Oxalobacter formigenes, which metabolize oxalate in the gut, does not lower the level of urine oxalate [15]. Corticosteroid treatment for patients with severely elevated serum creatinine levels showed no difference comparing the rate of kidney function recovery to patients without corticosteroid treatment [11]. If dialysis is required for severe cases, high-flux hemodialysis can be used for the removal of excess oxalate [13]. The overall prognosis of chronic ON remains poor, with a high rate of kidney failure, whereas preexisting CKD appears to be a risk factor for worse kidney outcomes [2]. Because targeted therapy is lacking, identifying such patients early to initiate the correct therapeutic steps, especially dietary measures and close follow-up care, is even more important.

## Conclusion

This case report provides a biopsy-proven case of fulminant acute renal failure in the context of acute oxalate nephropathy in a previously kidney-healthy patient, likely triggered by a single meal containing a large amount of freshly harvested chard, in the setting of a chronically oxalate-rich diet, minor volume depletion, and pre-existing therapy with ACEi, which may have contributed to renal consequences of acute oxalate excess. The acute episode may reflect a cumulative process of oxalate deposition, with the chard meal acting as a superimposed precipitating event that exceeded renal compensatory capacity. This case highlights the need for a detailed anamnesis for a suspicion of such a diagnosis in times when vegetarian and vegan diets, encompassing high oxalate ingestion, gain growing popularity. Nevertheless, the diagnosis of oxalate nephropathy can be confirmed only by a kidney biopsy for rapid initiation of conservative therapy, which involves the avoidance of further oxalate intake to preserve kidney function. This case highlights the importance of nutritional counselling, which led to a return to baseline eGFR just one year after acute ON.

## Data Availability

No datasets were generated or analysed during the current study.

## Declarations

### CARE guidelines

This case report has been prepared in accordance with the CARE guidelines.

## Abbreviations

ACEi: Angiotensin-converting enzyme inhibitor
ARF: Acute renal failure
CVA: Costovertebral angle
EH: Enteric hyperoxaluria
ON: Oxalate nephropathy
PH: Primary hyperoxaluria
